# Suppression of Cell Growth, Migration and Drug Resistance by Ethanolic Extract of *Antrodia cinnamomea* in Human Lung Cancer A549 Cells and C57BL/6J Allograft Tumor Model

**DOI:** 10.3390/ijms19030791

**Published:** 2018-03-09

**Authors:** Chi-Han Wu, Fon-Chang Liu, Chun-Hsu Pan, Ming-Tsung Lai, Shou-Jen Lan, Chieh-Hsi Wu, Ming-Jyh Sheu

**Affiliations:** 1 School of Pharmacy, China Medical University, Taichung 40402, Taiwan; u101055003@cmu.edu.tw (C.-H.W.); fonchang008@gmail.com (F.-C.L.); 2 School of Pharmacy, Taipei Medical University, Taipei 11031, Taiwan; panch@tmu.edu.tw (C.-H.P.); chhswu@tmu.edu.tw (C.-H.W.); 3 Department of Pathology, Taichung Hospital, Ministry of Health and Welfare Taiwan, Taichung 40343, Taiwan; mtlailuke@gmail.com; 4 Department of Healthcare Administration, Asia University, Taichung 41354, Taiwan; sjlan@asia.edu.tw

**Keywords:** *Antrodia cinnamomea*, anti-migration, anti-proliferation, paclitaxel resistance, lung cancer

## Abstract

The purpose of this study was to investigate the inhibitory activities of ethanolic extracts from *Antrodia cinnamomea* (EEAC) on lung cancer. Cell proliferation and cell cycle distribution were analyzed using (3-(4,5-Dimethylthiazol-2-yl)-2,5-diphenyltetrazolium bromide) (MTT) assay and flow cytometry, respectively. Wound-healing assay, Western blotting, and a murine tumor model were separately used to examine cell migration, protein expression, and tumor repression. Our results showed that EEAC induced cell cycle arrest at the G0/G1 phase resulting decreased cell viability in A549 cells. Moreover, EEAC up-regulated the growth-suppressing proteins, adenosine 5′-monophosphate-activated protein kinase (AMPK), p21 and p27, but down-regulated the growth-promoting proteins, protein kinase B (Akt), mammalian tarfet of rapamycin (mTOR), extracellular signal-regulating kinase 1/2 (ERK1/2), retinoblastoma protein (Rb), cyclin E, and cyclin D1. EEAC also inhibited A549 cell migration and reduced expression of gelatinases. In addition, our data showed that tumor growth was suppressed after treatment with EEAC in a murine allograft tumor model. Some bioactive compounds from EEAC, such as cordycepin and zhankuic acid A, were demonstrated to reduce the protein expressions of matrix metalloproteinase (MMP)-9 and cyclin D1 in A549 cells. Furthermore, EEAC enhanced chemosensitivity of A549 to paclitaxel by reducing the protein levels of caveolin-1. Our data suggests that EEAC has the potential to be an adjuvant medicine for the treatment of lung cancer.

## 1. Introduction

In general, the five-year survival rate of the patients with lung cancer is only 15% [1]. Non-small cell lung cancer (NSCLC), a major histological type of lung cancer, shows lower susceptibility to traditional chemotherapy and radiation treatments. Therefore, a more effective agent should be investigated.

For treatment of tumor metastasis, cell cycle arrest and migration inhibition are the targets of drug development. Several molecules are involved in the regulation of cell cycle and cell growth, including cyclin-dependent kinase (CDKs) and CDK inhibitors (CDKi) [2,3]. Additionally, AMP-activated protein kinase (AMPK), a cellular energy sensor, is involved in the control of tumor growth via activating p53-/p21 cascade and inhibiting the mTOR-mediated pathway [4]. Besides, Akt protein, known as an upstream regulator of mTOR, has been shown to stimulate cell growth and migration as well as to provide anti-apoptotic activity for cancer survival [5].

Moreover, matrix metalloproteinases (MMPs), are highly expressed in various types of human cancers and play a critical pathological role in tumor growth, metastasis and angiogenesis [6]. The extracellular signal-regulated kinase (ERK) associated with regulation of MMP-2 and MMP-9 proteins has been reported [7]. Furthermore, chemotherapy resistance is also associated with clinical unresponsiveness in cancer patients who receive chemotherapy drugs. Ho et al. showed that up-regulation of caveolin-1 was correlated with drug-resistant and poor progression-free survival rates in NSCLC patients [8]. Silencing of *cav-1* has been evidenced to enhance doxorubicin-induced apoptosis and reduced lung metastasis in human renal cell carcinomas [9].

*Antrodia cinnamomea* (*A. cinnamonea*) is a medicinal fungus that only grows inside the rotten trunk of *Cinnamomum kanehirae*, a native tree species of Taiwan [10]. *A. cinnamomea* has been explored to evaluate its effect in different cancers or use of adjuvant medicine for chemotherapy [11,12]. Our previous studies identified two main constituents, zhankuic acid A and cordycepin, in ethanolic extracts of *A. cinnamomea* (EEAC) by HPLC/Mass-fingerprint analysis [13]. The present study attempted to evaluate the mechanisms of anti-cancer activities and synergistic effects of the EEAC in A549 human lung adenocarcinoma epithelial cells and a C57BL/6J allograft tumor model.

## 2. Results

### 2.1. EEAC Induced Cell-Cycle Arrest and Reduced Cell Viability of A549 Cells

**Figure 1:**
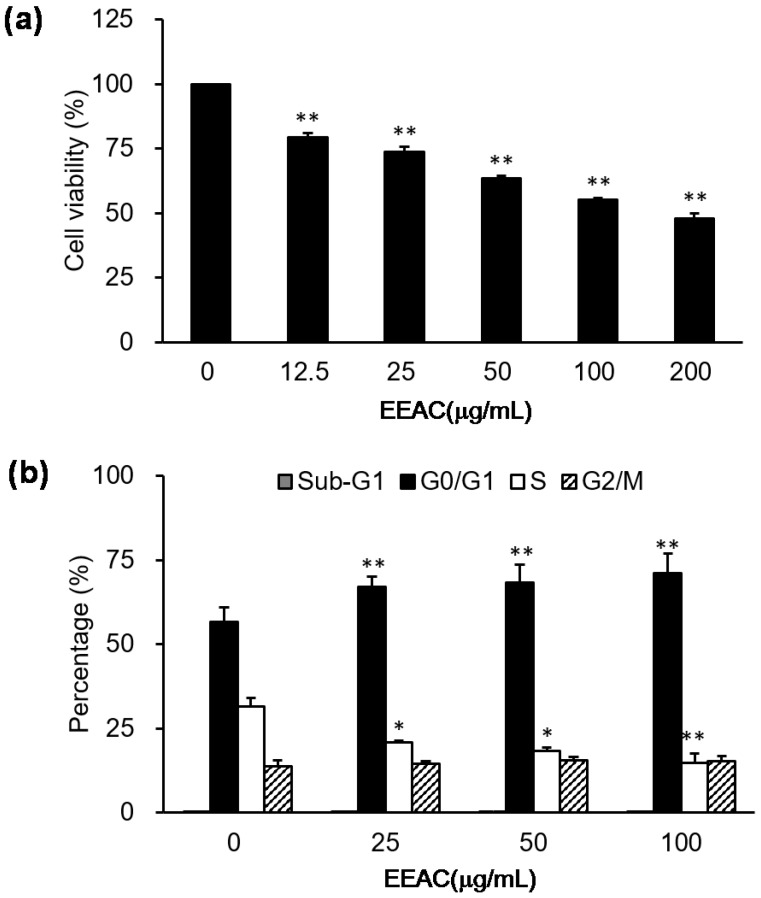
Growth regulation of ethanolic extracts from *Antrodia cinnamomea* (EEAC) in A549 cells. Cell viability and cell cycle distribution were, respectively, measured using an (3-(4,5-Dimethylthiazol-2-yl)-2,5-diphenyltetrazolium bromide) (MTT) assay (**a**) and a flow cytometer (**b**) in A549 cells treated with various concentrations of EEAC for 24 h.* *p* < 0.05 and ** *p* < 0.01 compared to the control group (without EEAC treatment), respectively.

Our results showed that various doses (12.5, 25, 50, 100, and 200 µg/mL) of EEAC reduced serum-stimulated cell growth of A549 cells in a dose-dependent manner (Figure 1a), and IC_50_ value of EEAC on A549 cells after a 24 h treatment was approximately 170 μg/mL. Moreover, the results obtained from flow cytometry demonstrated that growth inhibition of EEAC may be partially mediated by cell-cycle arrest at G0/G1 phase (Figure 1b). Specifically, the proportion of cells in the G0/G1 phase increased from 56% (control group) to 66% (25 µg/mL), 68% (50 µg/mL), and 71% (100 µg/mL).

### 2.2. Regulation of EEAC on Cell Growth-Associated Proteins in A549 Cells

**Figure 2:**
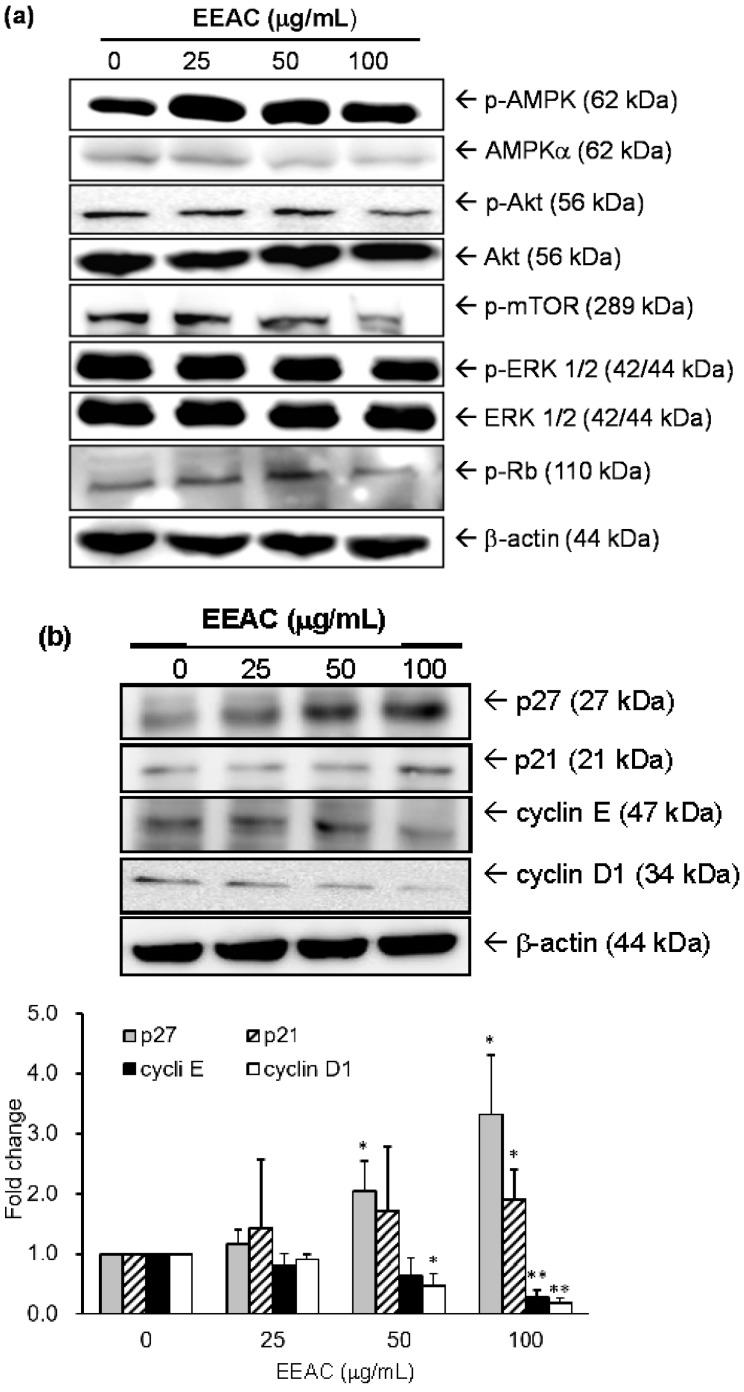
Effect of EEAC on cell growth-associated proteins in A549 cells. Cells were treated with several concentrations of EEAC for 30 min to examine the expression and/or activation levels of AMPKα, Akt, mTOR, and ERK1/2 (**a**). Each value represents the average of three independent experiments in Table 1. Protein expressions of p21, p27, cyclin D1 and cyclin E were incubated with the indicated concentrations of EEAC for 24 h (**b**), and fold changes of individual proteins were shown as a histogram. * *p* < 0.05 and ** *p* < 0.01 compared to the control group (treated with vehicle alone), respectively.

Several critical molecules involved in the regulation of cell growth were examined to understand the growth-inhibitory mechanisms of EEAC on A549 cells. Experimental data indicated that EEAC significantly increased the phosphorylation level of a growth-suppression protein, AMPKα, as well as dose-dependently inhibited activations of several growth-promoting proteins, such as Akt, mTOR, ERK1/2 and Rb. However, EEAC did not influence the total protein levels of these proteins (Figure 2a and Table 1). Furthermore, the cell cycle regulatory proteins, such as p27, p21, cyclin E, and cyclin D1, were also examined in A549 cells treated with EEAC for 24 h. The protein levels of cyclin E and cyclin D1 were reduced, while the p21 and p27 protein levels were increased in A549 cells with EEAC treatment (Figure 2b).

### 2.3. EEAC Suppressed Cell Migration of A549 Cells and Gelatinase Expression

**Figure 3:**
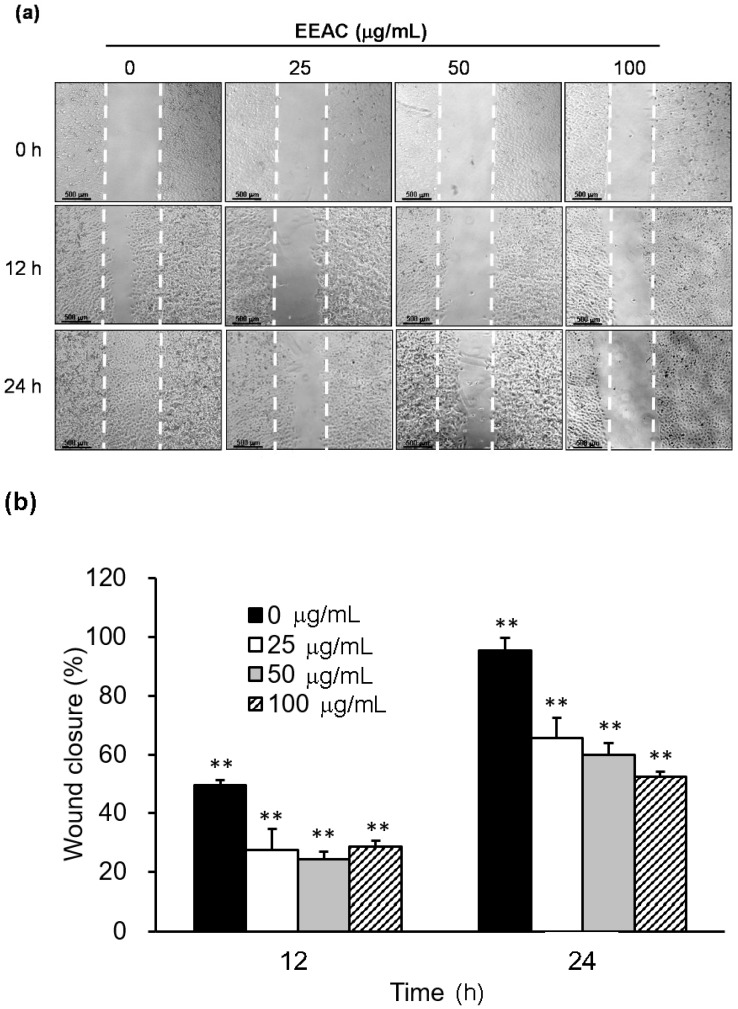
Inhibition of cell migration by EEAC in A549 cells. Cells were stimulated with 10% FBS to induce cell migration and co-incubated with various concentrations of EEAC. The pictures were acquired after 0, 12 and 24 h treatment of EEAC (**a**). Migration inhibition was presented as the percentage of wound closure by calculating the cell-free gap of each group compared to those at initial time (**b**). ** *p* < 0.01 compared to those of individual group at initial time.

**Figure 4:**
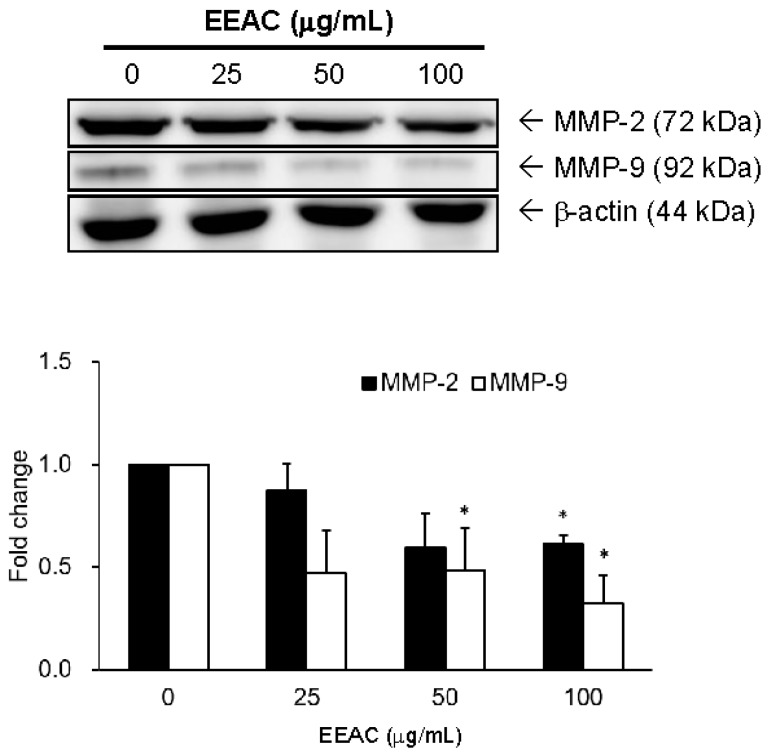
Suppression effect of EEAC on gelatinases in A549 cells. The expression levels of gelatinases, MMP-2 and MMP-9, were examined in A549 cells incubated with various concentrations of EEAC for 24 h. The band intensities of each group were quantified and normalized to the control group, and the relative expressions of gelatinases were presented as fold change in a histogram. * *p* < 0.05 compared to the control group (treated with vehicle alone).

Our results showed that serum stimulated cell migration of A549 cells, and this stimulation can be markedly reduced by EEAC (25, 50, and 100 µg/mL) incubation in a dose- and time-dependent manner (Figure 3). Additionally, the protein levels of gelatinases (MMP-2) were down-regulated from 100% (control group) to 87% (25 µg/mL), 60% (50 µg/mL), and 60% (100 µg/mL) in A549 cells after 24 h treatment of EEAC (Figure 4). Moreover, MMP-9 were downregulated from 100% to 47% (25 µg/mL), 48% (50 µg/mL), and 32% (100 µg/mL) in A549 cells after 24 h treatment of EEAC (Figure 4).

### 2.4. Zhankuic Acid A and Cordycepin Decreased Cyclin D1 and MMP-9 Expressions in A549 Cells

**Figure 5:**
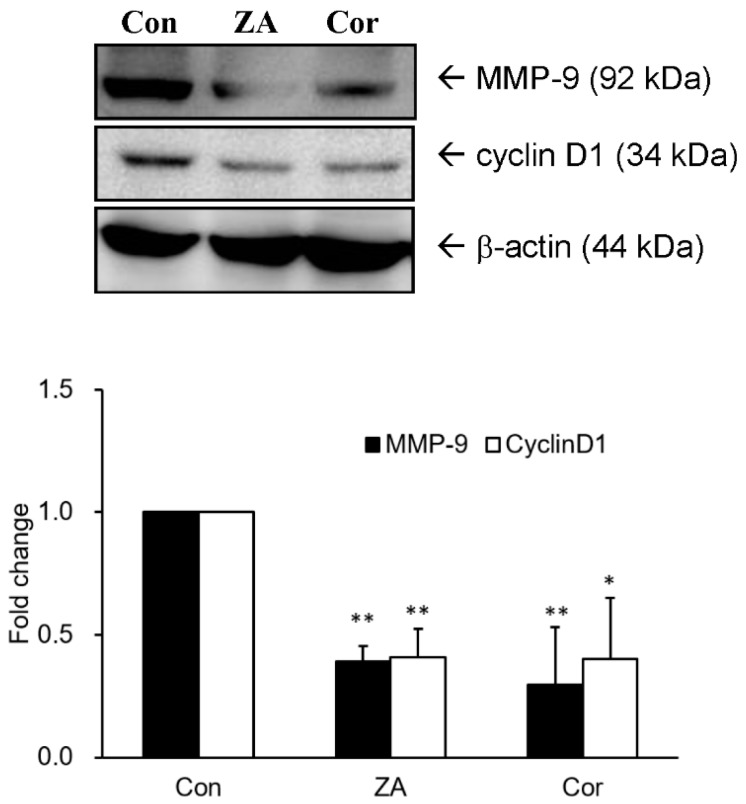
Effects of zhankuic acid A and cordycepin on the protein expressions of cyclin D1 and MMP-9 in A549 cells. The expression levels of cyclin D1 and MMP-9 were examined in A549 cells incubated with zhankuic acid A or cordycepin for 24 h. The band intensities of each group were quantified and normalized to the control group, and the relative expressions of gelatinases were presented as fold change in a histogram. * *p* < 0.05 and ** *p* < 0.01 compared to the control group (treated with vehicle alone), respectively. Con, control; ZA, zhankuic acid A; Cor, cordycepin.

Our investigation showed that expression levels of cyclin D1 and MMP-9 were markedly reduced in A549 cells after 24 h treatment with the bioactive compounds, zhankuic acid A (7.2 µM) and cordycepin (9.1 µM), identified in EEAC (Figure 5).

### 2.5. Regulation of EEAC on Chemosensitivity of A549 Cells to Paclitaxel

**Figure 6:**
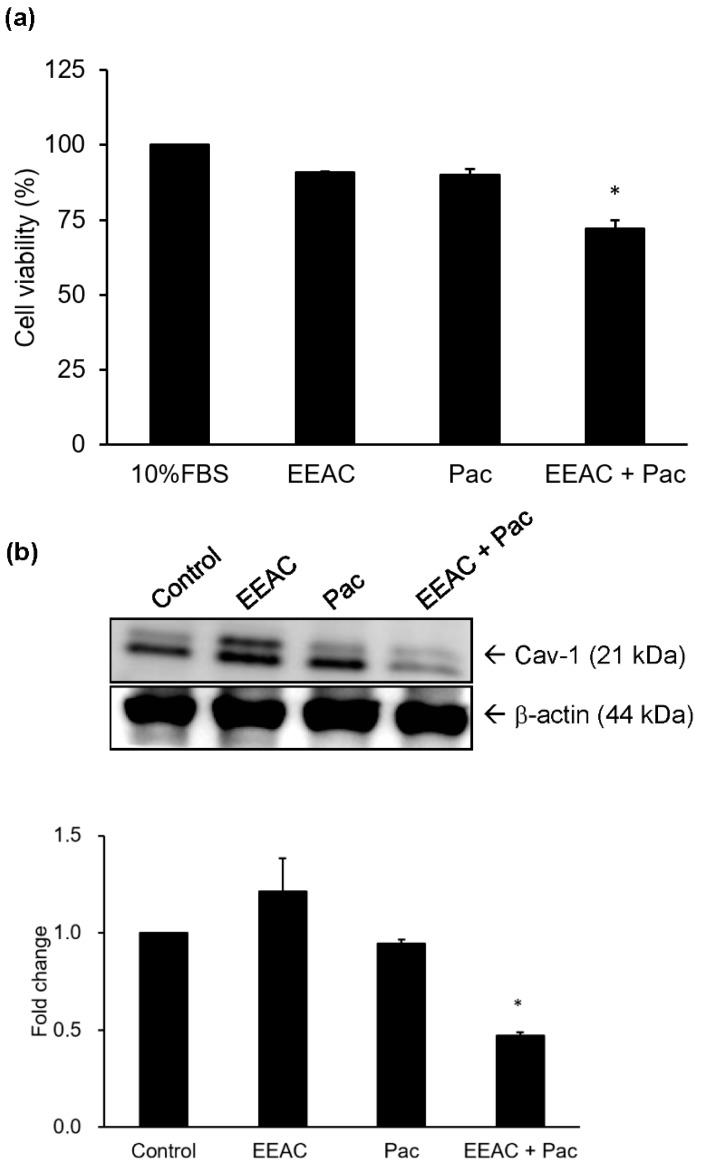
EEAC enhanced chemosensitivity of A549 cell to paclitaxel. Cells were incubated with EEAC (6.25 μg/mL), paclitaxel (0.156 μM; Pac) or EEAC combined with paclitaxel for 24 h to examine the change of cell viability (**a**). Under the same experimental condition, the expression level of cav-1 protein was examined using Western blot (**b**). The band intensities of each group were quantified and normalized to the control group, and the relative expression of cav-1 protein was presented as fold change in a histogram. * indicates *p* < 0.05 as compared with paclitaxel alone group. Pac, paclitaxel; Cav-1, caveolin-1.

Chemotherapy sensitivity of A549 cells to paclitaxel (Pac) was evaluated by using cell viability assays. Cells were treated with EEAC (6.25 μg/mL), Pac (0.156 μM) or a combination of EEAC and Pac. In order to evaluate the synergic inhibitory effect of EEAC and Pac on cell growth of A549 cells, both concentrations of EEAC and Pac used in this experiment were the IC_10_ value. Data showed that the combination group exhibited an enhanced chemotherapy effect by decreasing cell viability by 15% as compared to the group treated with Pac alone (Figure 6a). Moreover, the combination group had significantly decreased protein expression levels of caveolin-1 (Cav-1) as compared to the paclitaxel group (Figure 6b).

### 2.6. Effect of EEAC on the Expressions of Cyclin D1 and MMP-9 in LLC Cells

**Figure 7:**
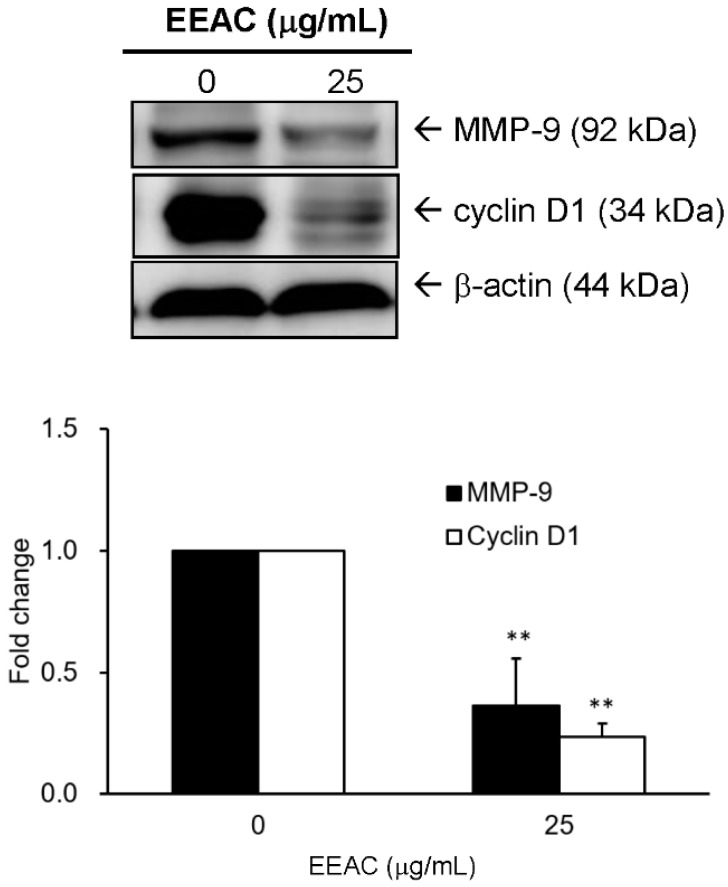
Effect of EEAC on cyclin D1 and MMP-9 expressions in LLC cells. Cells were incubated with 25 μg/mL EEAC for 24 h to analyze translational levels of cyclin D1 and MMP-9 proteins. The band intensities of each group were quantified and normalized to the control group (treated with vehicle alone), and the relative expressions of detected proteins were presented as fold change in a histogram. ** indicates *p* <0.01 as compared with control group, respectively.

To investigate whether EEAC affects regulators involved in the cell cycle and migration in LLC cells, cells were treated with EEAC (25 μg/mL) for 24 h. Experimental result indicated that the protein levels of MMP-9 and cyclin D1 were markedly reduced to 33% and 26%, respectively, after EEAC treatment (Figure 7).

### 2.7. EEAC Reduced Tumor Growth in the Lewis Lung Carcinoma Allograft Mode

**Figure 8:**
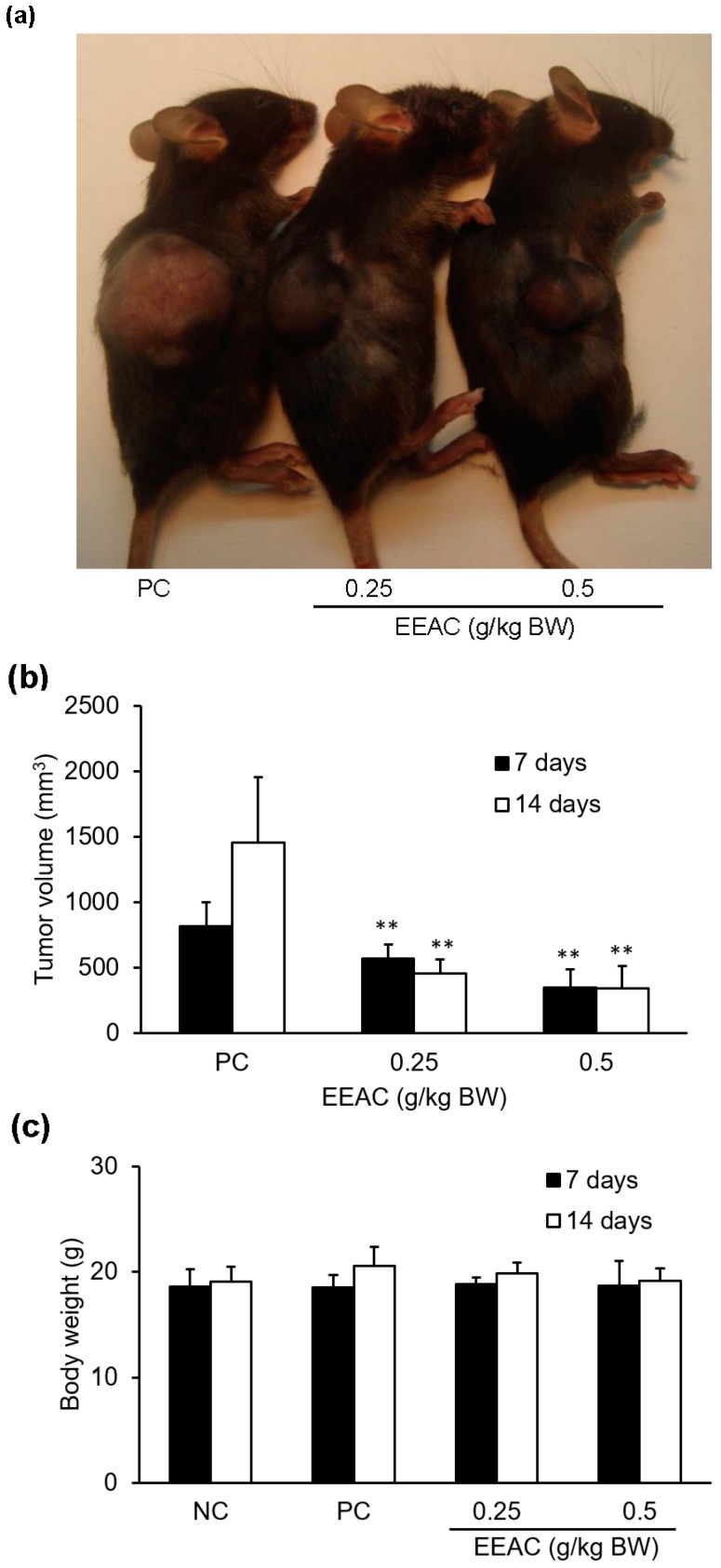
EEAC inhibited tumor growth in the Lewis lung carcinoma allograft model. The tumor tissue denoted by a dashed line was photographed at the end of the study. (**a**) Tumor volume (**b**) and bodyweight (**c**) of each group are shown as a histogram. NC: normal control, PC: LLC group. ** *p* < 0.01 compared to the PC group (treated with vehicle alone).

The results showed that a seven-day treatment of EEAC (0.25 and 0.5 g/kg) markedly reduced tumor size by 30% and 58%, respectively, as compared to the PC group (treated with vehicle alone; Figure 8a,b). These results also showed that a 14-day treatment of EEAC (0.25 and 0.5 g/kg) obviously reduced tumor size by 68% and 76%, respectively, as compared to the PC group. However, the body weights between EEAC group and PC group did not show significantl differences after seven or 14 days of oral administration (Figure 8c).

## 3. Discussion

Several bioactive compounds from EEAC, such as antroquinonol, 4-acetylantroquinonol B and zhankuic acid A, have been reported to be effective in reducing cell proliferation of hepatoma, colon cancer and leukemia cells [14,15,16]. Our previous study has identified three compounds including, adenosine, cordycepin and zhankuic acid A from EEAC [13]. Of these compounds, the G2/M arrest of cordycepin, which regulated cyclin D1, cyclin B1 and p21 protein, has been investigated in melanoma, bladder and colon cancer cells [17,18,19,20,21]. Besides, Chen et al. reported that zhankuic acid A exhibited cytotoxic activity against P-388 murine leukemia cells [22]. These may be due to regulation of CDKs and CDKIs and subsequent blocking of cell cycle progression [23,24]. In addition, studies have reported that cyclin D1, cyclin E, MMP-2 and MMP-9 are associated with tumor growth and metastasis inhibition in an A549 cell and its xenograft mouse tumor model [25,26,27].

In our previous study, cordycepin (0.16 mg/g) and zhankuic acid A (235 mg/g) were found in EEAC, respectively [13]. Thus, in accordance with concentrations found in the former, the 100 μg/mL EEAC solution were used to investigate their effect on cyclin D1 protein expression in the present study. After treatment with cordycepin and zhankuic acid A, cyclin D1 protein levels were markedly decreased in A549 cells (Figure 5). This may partially explain how these two major constituents of EEAC are involved in the regulation of A549 cell cycle arrest, which correlated with the decreasing cell viability and inducing G0/G1 and S phases arrest (Figure 1).

Both of p21 and p27 are known to regulate G1/S and G2/M transitions [28]. Niculescu et al. also provided evidence that p21 can regulate the phosphorylation of Rb and the subsequent blocking of DNA replication to prevent endoreduplication in different cancer cells at both the G1/S and the G2/M cell cycle transitions [29]. Moreover, p27-deficiency enhances chronic hepatocyte injury-induced liver tumorigenesis by overexpression of cyclin E1, which contributes to the activation of cdk2 [30]. Our results demonstrated that the protein levels of p21 and p27were increased, and the levels of cyclin D1 and cyclin E were decreased in A549 cells after treatment with EEAC (Figure 2b).

Many studies indicated AMPK dysfunction increases the viability of lung cancer [31]. Our results indicated that EEAC not only increased the activation of AMPKα but also decreased Akt and mTOR expression in A549 cells (Figure 2a and Table 1). This may be attributed to the regulations of zhankuic acid A and cordycepin in AMPK and Akt [32,33]. Several studies showed that Akt and ERK1/2 signaling pathways modulate cell migration and invasion by regulating MMP expression in NSCLC [34,35]. Besides, more than 34% of lung tumor tissues of NSCLC patients were observed to have higher levels of activated ERK1/2 as compared with normal tissue sections [36], indicating advanced and aggressive NSCLC tumors. A study also revealed that inactivation of ERK1/2 was found to inhibit the migration and invasion of lung cancer cells [37]. In our study, the decrease of reported cell migration can be observed after administrating EEAC (Figure 3). The results showed that the phosphorylations ofAkt and ERK1/2 as well as the protein expressions of MMP-2 and 9 were significantly decreased which is similar to what was reported in previous studies (Figure 2a and Figure 4).

Chemotherapy remains a standard remedy for advanced cancer. Pac, a valuable cancer chemotherapeutic agent, is widely applied in the treatment of many metastatic types of cancer, including ovary, breast, and lung carcinomas. However, cancer cells may also be resistant to Pac, which causes failure during treatments and increased mortality, and this turns out to be a major clinical concern. Cav-1 is a membrane-associated protein with two isoforms, caveolin-1α and β, that is not only a putative regulator of cellular transformation but also a regulator of the activity of survival-associated proteins, such as Src kinases, epidermal growth factor tyrosine kinase, Her2/neu (ErbB2) kinase, ERK, endothelial nitric-oxide synthase, and G proteins [38]. Cav-1 also inhibits serine/threonine protein phosphatases (PP1 and PP2A). This inhibition leads to an up-regulation of Akt and ERK1/2 activation and subsequently induces cancer cell growth [39]. Moreover, silencing of the *cav-1* gene enhanced doxorubicin-induced apoptosis and reduced lung metastasis in human renal cell carcinoma [40]. Our results suggest that using non-toxic Pac combined with EEAC reduced the protein expression of cav-1 compared to applying Pac alone (Figure 6a,b). Pac co-treatment with EEAC exhibited enhanced chemosensitivity of A549 cells. These results indicate that EEAC could be an adjuvant remedy to alleviate the clinical problem of paclitaxel resistance in NSCLC.

According to the present study, A549 and LLC cells showed regulation of protein levels related to cell cycle and migration in response to EEAC treatment (Figure 1, Figure 2, Figure 4, and Figure 7). Therefore, we used an C57BL/6J mice allograft tumor model to evaluate the anticancer effect of EEAC and the results showed that EEAC significantly decreased the average tumor volume of C57BL/6J mice injected with Lewis lung cancer cells without affecting mice body weight (Figure 8).

In conclusion, our investigation demonstrated that EEAC has effective inhibitory activities on cell growth, migration and paclitaxel resistance ofA549 cancer cells. These pharmacological effects might be partially mediated through inhibiting gelatinases to reduce migratory ability as well as by suppressing the growth-promoting proteins, Akt, ERK1/2, cyclin D1, and cyclin E, and by the activating growth-suppressing proteins, p-AMPK, p21 and p27, to decrease cell growth. EEAC also decreased the protein expression of cav-1 to enhance the cytotoxicity of paclitaxel in A549 cells. The result obtained from the murine allograft tumor model indicated that EEAC has anti-tumor activity. Therefore, EEAC has potential for development as an adjuvant drug for clinical chemotherapy of lung cancer.

## 4. Materials and Methods

### 4.1. Materials

Fetal bovine serum (FBS), penicillin G, Dulbecco’s modified Eagle’s medium (DMEM), Ham’s F12K medium, and streptomycin were obtained from Invitrogen (Carlsbad, CA, USA). Sodium pyruvate and non-essential amino acids were purchased from Biological Industries (Kibbutz Beit Haemek, Israel). Primary antibodies against p-Rb (#sc-16670), p-ERK1/2 (#sc-7383) and ERK1/2 (#sc-154) were purchased from Santa Cruz Biotechnology (Santa Cruz, CA, USA). Antibodies recognized p-AMPK (#2535), caveolin-1 (#3238), p-mTOR (#2971) and AMPKα (#2793) were obtained from Cell signaling Technology (Beverly, MA, USA). Antibodies against Akt (ab28422), p-Akt (#ab28821), MMP-2 (#ab37150), MMP-9 (#ab58803), p21 (#ab379601) and β-actin (#ab8226) were from Abcam (Cambridge, MA, USA).Anti-p27 (#GTX100446), Anti-cyclin D1 (#GTX27958), and Anti-cyclin E (#GTX27959) antibodies were obtained from GeneTex (Irvine, CA, USA). Secondary antibodies conjugated with horseradish peroxidase (HRP) were obtained from Santa Cruz Biotechnology. The zhankuic acid A was isolated, purified and identified according to our previous study [41]. Other reagents were purchased from Sigma-Aldrich (St. Louis, MO, USA).

### 4.2. Preparation of Ethanolic Extract of Antrodia cinnamomea (EEAC)

The extraction procedure of EEAC was performed according to our previous study [42]. A stock solution EEAC was prepared as 100 mg/mL by 95% ethanol for in vitro studies. For in the vivo study, EEAC was firstly dissolved in 95% ethanol to give a 650 mg/mL stock solution that was further diluted to 65 mg/mL by PBS solution with 0.5% (*v*/*v*) Tween-80.

### 4.3. Cell Culture

A549 cells (#BCRC-60074), a human lung adenocarcinoma cell line, and LLC cells (#BCRC-60050), a mouse Lewis lung carcinoma, were obtained from the Bioresource Collection and Research Center (BCRC) of the Food Industry Research and Development Institute (Hsinchu, Taiwan). A549 cells were cultured in Ham’s F12K medium supplemented with 10% fetal bovine serum (FBS; #10099-141, Invitrogen), 1.5 g/L sodium bicarbonate, 2 mM l-glutamine, 100 units/mL penicillin G and 100 μg/mL streptomycin sulfate. LLC cells were maintained in DMEM medium containing 10% FBS, 1.5 g/L sodium bicarbonate, 4 mM l-glutamine, 4.5 g/L glucose, 100 units/mL penicillin G and 100 μg/mL streptomycin sulfate. All cells were incubated in a humidifier with 5% CO_2_ at 37 °C, and the culture medium was refreshed every 2 days. 

### 4.4. Cell Viability Assay (MTT Assay)

A549 cells (2 × 10^4^ cells/well) were seeded into a 96-well overnight. A549 cells were exposed to 100 μL of different concentrations of the tested drugs (e.g., EEAC or paclitaxel) in culture medium. After 24 h of treatment, 10 μL of 5 mg/mL MTT (3-(4,5-dimethylthiazol-2-yl)-2,5-diphenyl tetrazolium bromide) was added into each well. After 4 h incubation, cells were washed twice with 1× PBS, and then 200 μL of dimethyl sulfoxide (DMSO) was added to each well. Absorbance values at 570 nm were determined for each well using 650 nm as the reference wavelength. The absorbance can be correlated to the percentage of vital cells by comparison with the control group (without treatment of tested drugs). The cell viability ratio was calculated by the following formula: cell viability (%) = OD (treated)/OD (control) × 100%.

### 4.5. Cell Cycle Analysis

EEAC-treated cells were harvested, washed twice with cold 1× PBS, and then fixed with chilled 70% ethanol overnight. After ethanol was removed by centrifugation at 1500 rpm for 15 min at 4 °C, the cell pellets were re-suspended in 500 μL of DNA staining buffer containing 4 μg/mL of propidium iodide, 1% Triton X-100 and 0.1 mg/mL RNase A and incubated for 30 min at room temperature in the dark. The cell cycle profile was analyzed using FACSCanto system (BD Biosciences, San Jose, CA, USA). Post-acquisition data analysis was performed using ModFit LT software (Verify Software House, Topsham, ME, USA).

### 4.6. Wound-Healing Analysis

Cells (2.5 × 10^4^ cells/well) were seeded into 24-well plates overnight. After 24 h of starvation, a cell-free gap was created with a 200 μL micropipette tip and then the cells were treated with various concentrations of EEAC in normal culture medium. Photographs of the cell-free gap were taken at 0, 12 and 24 h after treatment, and the area of the cell-free gap was measured by Image J software (NIH, Bethesda, MD, USA). Migration inhibition was presented as the percentage of wound closure by calculating the cell-free gap of each group compared to those at the initial time (0 h).

### 4.7. Western Blot Analysis

Harvested cells were lysed by PRO-PREP Protein Extraction Kit (iNtRON Biotechnology, Gyeonggi-do, Korea) 5and centrifuged at 10,000 rpm at 4 °C for 30 min. The supernatant was incubated at 95 °C for 5 min in 6× sample loading buffer containing 0.35 M Tris-HCl (pH 6.8), 10% SDS, 30% glycerol, 0.12% bromophenol blue and 6% β-mercaptoethanol. About 50 μg of total protein was separated by 10% sodium dodecyl sulfate-polyacrylamide gel electrophoresis (SDS-PAGE) followed by blotting to a polyvinylidene fluoride (PVDF) membrane (NEF1002001PK; Perkin Elmer, Boston, MA, USA). Non-specific binding of the blotted membrane was blocked by using 5% non-fat milk dissolved in 1× Tris-buffered saline (TBS) with 0.2% Tween 20 (0.2% TBST). The membrane was incubated with the primary antibody (in 0.2% TBST) overnight at 4 °C. Subsequently, the secondary antibody (in 0.2% TBST) was applied for 3 h at 4 °C. The expected protein bands were visualized using the ECL reaction (Amersham, Arlington Height, IL, USA), and the luminescence signal was acquired with the Fujifilm LAS-4000 system (San Leandro, CA, USA). Band intensity was analyzed using MultiGauge software (Fujifilm). The band intensity of individual proteins was normalized to that of β-actin and presented as the fold change from the control group (without the treatment of tested drugs).

### 4.8. Lewis Lung Cancer Allograft Model in C57BL/6J Mice

Male C57BL/6J mice (6 weeks old) were obtained from BioLASCO (Taipei, Taiwan). The C57BL/6J mice were housed in the Laboratory Animal Center, China Medical University (Taichung, Taiwan) and kept on 12 h light/dark cycles at 25 °C. The animal experiments were approved by the Institutional Animal Care and Use Committee of China Medical University (IACUC; approval ID: 102-194-C, approval date: 04-01-2013). All animal care followed the institutional animal ethical guidelines of China Medical University. After adaptation for 1 week, C57BL/6J mice were randomly divided into subcutaneously implanted with LLC cells (7 × 10^5^ cells in 100 μL of cell culture medium). After 2~3 weeks of tumor induction, mice were randomly divided into four groups (*n* = 6/group): Group Ι (normal control group, NC) was given vehicle solvent. Group ΙI (untreated LLC group, PC) was grafted with LLC cells and given vehicle solvent. Group III was grafted with LLC cells and then treated with EEAC (0.25 g/kg). Group IV was grafted with LLC cells and then treated with EEAC (0.5 g/kg). The EEAC and vehicle solvent was administrated by oral gavage once a day for 2 weeks. Tumor width (W) and length (L) were measured by calipers every week to calculate the tumor volume by the formula: L × W^2^ × 0.52 [16]. Body weight was also recorded every week during the study.

### 4.9. Statistical Analysis

All data are presented as mean ± standard deviation (S.D.). All experiments were done in triplicate. Statistical significance was evaluated by one-way ANOVA with a Bonferroni post-hoc test. A value of *p* < 0.05 was regarded as being statistically significant.

## Figures and Tables

**Table 1:** Fold changes of detected proteins in A549 cells treated with EEAC.

Protein Name	EEAC (μg/mL)			
	0	25	50	100
p-mTOR	1	0.78 ± 0.36	0.85 ± 0.37	0.43 ± 0.10 **
p-Rb	1	0.87 ± 0.17	0.86 ± 0.01 *	0.39 ± 0.06 **
p-AMPK	1	2.42 ± 0.02 **	2.49 ± 0.11 **	2.49 ± 0.51 *
AMPK	1	1.89 ± 0.28	1.57 ± 0.35	1.51 ± 0.46
p-Akt	1	1.04 ± 0.23	1.00 ± 0.17	0.74 ± 0.06 *
Akt	1	1.59 ± 0.27	1.72 ± 0.40	1.98 ± 0.72
p-ERK1/2	1	1.07 ± 0.23	0.80 ± 0.10	0.50 ± 0.11 *
ERK1/2	1	1.13 ± 0.02	0.93 ± 0.17	0.89 ± 0.05

* *p* < 0.05 and ** *p* < 0.01 compared to the control group (treated with vehicle alone), respectively. p-mTOR: Phospho-mammalian target of rapamycin; p-Rb: Phospho-retinoblastoma protein; p-AMPK: Phospho-AMP-activated protein kinase; AMPK: Adenosine 5′-monophosphate (AMP)-activated protein kinase; p-Akt: Phospho-protein kinase B; Akt: Protein kinase B; p-ERK1/2: Phospho-extracellular signal-regulating kinase 1/2; ERK1/2: Extracellular signal-regulating kinase 1/2.
